# Identification of BCL-XL as highly active survival factor and promising therapeutic target in colorectal cancer

**DOI:** 10.1038/s41419-020-03092-7

**Published:** 2020-10-17

**Authors:** Anna-Lena Scherr, Andreas Mock, Georg Gdynia, Nathalie Schmitt, Christoph E. Heilig, Felix Korell, Praveen Rhadakrishnan, Paula Hoffmeister, Klaus H. Metzeler, Klaus Schulze-Osthoff, Anna L. Illert, Melanie Boerries, Jörg Trojan, Oliver Waidmann, Johanna Falkenhorst, Jens Siveke, Philipp J. Jost, Michael Bitzer, Nisar P. Malek, Loredana Vecchione, Ivan Jelas, Benedikt Brors, Hanno Glimm, Albrecht Stenzinger, Svetlana P. Grekova, Tobias Gehrig, Henning Schulze-Bergkamen, Dirk Jäger, Peter Schirmacher, Mathias Heikenwalder, Benjamin Goeppert, Martin Schneider, Stefan Fröhling, Bruno C. Köhler

**Affiliations:** 1 Department of Medical Oncology, National Center for Tumor Diseases (NCT), University Hospital Heidelberg, 69120 Heidelberg, Germany; 2 Department of Translational Medical Oncology, National Center for Tumor Diseases (NCT) Heidelberg, German Cancer Research Center (DKFZ), 69120 Heidelberg, Germany; 3 German Cancer Consortium (DKTK), 69120 Heidelberg, Germany; 4 Institute of Pathology, University Hospital Heidelberg, 69120 Heidelberg, Germany; 5 Department of Medicine V, University Hospital Heidelberg, 69120 Heidelberg, Germany; 6 Department of General, Visceral and Transplantation Surgery, University of Heidelberg, 69120 Heidelberg, Germany; 7 Department of Medicine III, University Hospital, LMU Munich, 81377 Munich, Germany; 8 Department of Molecular Medicine, Interfaculty Institute for Biochemistry, University of Tübingen, 72076 Tübingen, Germany; 9 Department of Internal Medicine I, Medical Center—University of Freiburg, Faculty of Medicine, University of Freiburg, 79106 Freiburg, Germany; 10 German Cancer Consortium (DKTK) Partner Site Freiburg and German Cancer Research Center (DKFZ), 69120 Heidelberg, Germany; 11 Comprehensive Cancer Center Freiburg (CCCF), Medical Center—University of Freiburg, Faculty of Medicine, University of Freiburg, 79106 Freiburg, Germany; 12 Institute of Medical Bioinformatics and Systems Medicine, Medical Center—University of Freiburg, Faculty of Medicine, University of Freiburg, 79106 Freiburg, Germany; 13 Department of Medicine 1, University Hospital Frankfurt, 60590 Frankfurt, Germany; 14 Universitäres Centrum für Tumorerkrankungen (UCT), University Hospital Frankfurt, 60590 Frankfurt, Germany; 15 Depārtment of Medical Oncology, Sarcoma Center, West German Cancer Center, University Duisburg-Essen, Medical School, 45147 Essen, Germany; 16 DKTK partner site Essen and German Cancer Consortium (DKTK), 69120 Heidelberg, Germany; 17 Institute for Developmental Cancer Therapeutics, West German Cancer Center, University Hospital Essen, 45147 Essen, Germany; 18 Division of Solid Tumor Translational Oncology, German Cancer Consortium (DKTK, partner site Essen) and German Cancer Research Center, DKFZ, 69120 Heidelberg, Germany; 19 Medical Department III for Hematology and Oncology, School of Medicine, Klinikum rechts der Isar, Technical University of Munich, 81675 Munich, Germany; 20 Central Institute for Translational Cancer Research (Translatum), Technical University of Munich, 81675 Munich, Germany; 21 German Consortium for Translational Cancer Research (DKTK) partner site TUM, German Cancer Research Center Heidelberg (DKFZ), 69120 Heidelberg, Germany; 22 Department of Internal Medicine I, University Hospital Tübingen, 72076 Tübingen, Germany; 23 Charité Comprehensive Cancer Center, 10117 Berlin, Germany; 24 Department of Hematology, Oncology and Tumor Immunology (CCM) Charité - Universitaetsmedizin Berlin, 10117 Berlin, Germany; 25 Division of Applied Bioinformatics, German Cancer Research Center (DKFZ), 69120 Heidelberg, Germany; 26 Department of Translational Medical Oncology, National Center for Tumor Diseases (NCT) Dresden and German Cancer Research Center (DKFZ), 01307 Dresden, Germany; 27 Center for Personalized Oncology, University Hospital Carl Gustav Carus Dresden at TU Dresden, 01307 Dresden, Germany; 28 German Cancer Consortium (DKTK) Dresden, 01307 Dresden, Germany; 29 Department of General and Visceral Surgery, Spital Linth, 8730 Uznach, Switzerland; 30 Department of Internal Medicine II, Marien-Hospital, 46483 Wesel, Germany; 31 Division of Chronic Inflammation and Cancer, German Cancer Research Center (DKFZ), 69120 Heidelberg, Germany

**Keywords:** Cancer therapeutic resistance, Apoptosis, Colorectal cancer

## Abstract

Since metastatic colorectal cancer (CRC) is a leading cause of cancer-related death, therapeutic approaches overcoming primary and acquired therapy resistance are an urgent medical need. In this study, the efficacy and toxicity of high-affinity inhibitors targeting antiapoptotic BCL-2 proteins (BCL-2, BCL-XL, and MCL-1) were evaluated. By RNA sequencing analysis of a pan-cancer cohort comprising >1500 patients and subsequent prediction of protein activity, BCL-XL was identified as the only antiapoptotic BCL-2 protein that is overactivated in CRC. Consistently, pharmacologic and genetic inhibition of BCL-XL induced apoptosis in human CRC cell lines. In a combined treatment approach, targeting BCL-XL augmented the efficacy of chemotherapy in vitro, in a murine CRC model, and in human ex vivo derived CRC tissue cultures. Collectively, these data show that targeting of BCL-XL is efficient and safe in preclinical CRC models, observations that pave the way for clinical translation.

## Introduction

Colorectal cancer (CRC) ranks among the most frequent cancers worldwide and represents a leading cause of cancer-related death^1,2^. In the metastasized situation (UICC stage IV), chemotherapy remains the backbone of standard of care therapeutic approaches^3^. In addition to poly-chemotherapy, targeting EGFR and VEGFR complements therapeutic regimes in clinical practice^4^. In the attempt to reach a more personalized patient stratification, the mutational landscape including pan-RAS, BRAF, MSI-status as well as sidedness of the tumor is taken into account^3,5–8^. Even if systemic treatment of metastatic CRC underwent fundamental improvements, curation is not in sight. Thus, an evident clinical need to improve CRC treatment prompted us to study novel combinatorial approaches.

Primary treatment failure as well as acquired resistance is determined by defective cell death signaling^9–11^. In several studies it has been shown that cells accumulate proapoptotic proteins in the course of oncogenic transformation, caused by check point evasion and DNA damage response^12,13^. Nevertheless, a considerable percentage of tumor cells manages to escape from cell death induction by counterbalancing increased levels of proapoptotic proteins with upregulation of antiapoptotic factors^14–16^. In this context, the BCL-2 family of proteins plays an essential role^17^. The family comprises pro and antiapoptotic proteins opposing each other. Among antiapoptotic BCL-2 proteins, BCL-2 itself, BCL-XL and MCL-1 are most prominent. They sequester and thereby inhibit the proapoptotic multidomain BCL-2 proteins, BAX and BAK, which assemble to pores in the outer mitochondrial membrane to initiate the intrinsic pathway of apoptotic cell death^18^. A third group of proteins, the BH3-only proteins, contribute to apoptosis either by competing with BAX or BAK for the binding of antiapoptotic proteins or by directly activating them for cytochrome c release.

The vision of utilizing this delicate balance to drive tumor cells over the edge of apoptosis induction, led to the development of small-molecule inhibitors targeting antiapoptotic protein function (BH3-mimetics)^19^. The BCL-2 specific inhibitor venetoclax (ABT-199) was the first compound in this field that became approved by the United States Food and Drug Administration (FDA) for treatment of chronic lymphocytic leukemia (CLL) and selected acute myeloid leukemia (AML) patients^20–23^.

In contrast to hematologic malignancies, where BCL-2 seems most important for tumor cell survival, the response of solid tumors to antiapoptotic BCL-2 protein inhibition is far more complex, due to their heterogeneity. Amplification of the genes coding for MCL-1 and BCL-XL (*BCL2L1*) has been found to rank among the most common copy number alterations in various solid tumor entities^24^. In the context of CRC, especially BCL-XL has been identified as a driver of oncogenesis and tumor progression^25,26^. In a murine CRC model, we have previously shown that specific deletion of BCL-XL in the intestinal epithelium renders mice less susceptible towards chemically induced carcinogenesis via increasing apoptosis induction in tumor cells^25^.

Following our preceding studies, and equipped with new and highly specific BH3-mimetics, the aim of this study was to further delineate the potential of targeting antiapoptotic BCL-2 proteins, especially BCL-XL, in the context of CRC treatment in terms of efficacy, toxicity and as a combinatorial approach with standard-of care therapeutic agents.

## Methods and materials

### RNA sequencing and estimation of protein activity in the NCT/DKTK MASTER cohort

The MASTER (Molecularly Aided Stratification for Tumor Eradication Research) program of the NCT Heidelberg and the German Cancer Consortium (DKTK) is a registry trial and analytical platform for prospective, omics-driven stratification of younger adults with advanced-stage cancer across all histologies and patients with rare tumors^27^. As of September 2019, a total of 1521 patient samples, including 68 from patients with metastatic CRC, have been analyzed by RNA sequencing. Furthermore, the activity of 6014 proteins was inferred from RNA sequencing data by Master Regulator Analysis^28^ employing the metaVIPER algorithm^29^. The viper R package^30^ was used to estimate the protein activity in an entity-independent fashion by integrating regulatory networks from 24 different TCGA tumor entities. The regulatory networks were reverse engineered by the ARACNe (algorithm for the reconstruction of accurate cellular networks) algorithm^31^ and are available through the aracne.network R package^32^. Through the integration of interactomes, the activity of 6013 proteins that were adequately represented in at least one of the available regulatory networks could be inferred in the MASTER cohort.

### Cell lines and reagents

The human CRC cell lines Colo205, HT29, CaCo2 and SW480 as well as the nontransformed human colon cell line CCD 841 CoN were purchased from ATCC (Manassas Virginia, USA). CRC cells were maintained in RPMI + GlutaMAX medium (Gibco, Karlsruhe, Germany) and CCD 841 CoN cells in DMEM medium (Gibco), in each case supplemented with 10% heat-inactivated fetal calf serum (PAA Laboratories, Cölbe, Germany) and 1% penicillin/streptomycin (PAA Laboratories) and cultured in a humidified atmosphere at 37 °C and 5% CO_2_. Staurosporine and chemotherapeutic reagents 5-fluorouracil (5FU) and irinotecan were purchased from Sigma–Aldrich (Munich, Germany). Selective inhibitors ABT-199 (BCL-2 inhibitor), S63845 (MCL-1 inhibitor), and A-1331852 (orally available BCL-XL inhibitor, used for murine experiments) were purchased from Selleckchem (Munich, Germany) and WEHI-539 (BCL-XL inhibitor, used for in vitro and ex vivo experiments) was purchased from Hycultec (Beutelsbach, Deutschland).

### Protein isolation, SDS-PAGE and western blot analysis

For protein analysis, cells were seeded and treated as indicated. Protein isolation was performed as described previously^33^ and equal amounts of protein were separated by 12% SDS-PAGE and blotted onto nitrocellulose membranes by standard procedures. Immunoblotting was performed using the following primary antibodies: BCL-XL (# 2764, Cell Signaling Technology, Danvers, MA, USA), MCL-1 (# sc-819, Santa Cruz Biotechnology, Heidelberg, Germany), BCL-2 (# ab692, Abcam, Cambridge, UK), and Tubulin (# T8203, Sigma–Aldrich) as well as peroxidase-conjugated secondary antibodies (Santa Cruz Biotechnology). Bound antibody was visualized using an enhanced chemiluminescence detection system (Perkin Elmer, Zaventem, Belgium) and signal intensity was measured using ImageJ^®^ software (by Wayne Rasband at NIH, Bethesda, Maryland, USA).

### Caspase activity assay

Cells were seeded into white-walled 96-well plates with a clear and flat bottom. After 24 h of incubation, cells were treated with the indicated concentrations of WEHI-539 and incubated for another 48 h. For measuring the activities of Caspases 3/7, 8, and 9 the luminescence-based Caspase-Glo Assay Kits from Promega (Madison, WI, USA) have been used, according to the manufacturer´s protocol. Luminescence of each sample was quantified in a plate-reading luminometer (Infinite 200 PRO; Tecan, Männedorf, Switzerland).

### Quantification of cell death by flow cytometry

Cells were seeded and treated as indicated for 48 h. Subsequently, supernatants were transferred to FACS tubes and cells were gently detached using Accutase (PAA Laboratories) and transferred into the tubes as well. After centrifugation (200 × *g*, 5 min), cells were resuspended in a hypotonic buffer containing 0.1% (w/v) sodium citrate, 0.1% (v/v) Triton X-100 and 50 µg/ml propidium iodide (all from Sigma–Aldrich). After 1 h of incubation in the dark at 4 °C, total DNA content of cells was measured according to the protocol of Nicoletti et al. by FACS analysis^34^, using the FACS Diva 6 and the FlowJo 7.6.5. software (BD Biosciences, San José, CA, USA). Cells in the sub-G1 fraction were considered apoptotic.

### siRNA transfection

Cells were seeded, cultured for 24 h and transfected by using Lipofectamine RNAiMAX (Thermo Fisher Scientific, Waltham, MA, USA) in OptiMEM cell culture medium without any supplements (Gibco, Karlsruhe, Germany), according to the manufacturer´s protocol. The following siRNA sequence, targeting the BCL-XL (*BCL2L1*) mRNA was applied (Eurofins Scientific, Luxembourg): 5‘→3‘ GCUUGGGAUAAAGAUGCAA (sense) and 5‘→3‘ UUGCAUCUUUAUCCCAAGC (antisense). As a control served the following scrambled siRNA: 5‘→3‘ AGACCCACUCGGAUGUGAAGAGAUA (sense) and 5‘→3‘ UAUCUCUUCACAUCCGAGUGGGUCU (antisense).

### Mice and AOM/DSS model

C57BL/6 mice were purchased from Charles River Laboratories (Wilmington, MA, USA) and housed in individually ventilated cages at the SPF animal facility of the Interfaculty Biomedical Research Facility (IBF), University of Heidelberg, Germany. Animals were kept under a 12 h light cycle with ad libitum feeding and all experiments on mice were conducted according to institutional, national and European animal regulations with all protocols approved by local government authorities and a daily review of the health status of the animals was done. The health status was gathered using a scoring system, which comprised diarrhea severity, weight loss, rectal prolapse, apathy and aggressiveness as a sign for pain.

28 gender-matched mice (to account for possible drop-outs during tumor induction) with an age of ten weeks and a body weight >20 g were injected intraperitoneally with 10 mg per kg body weight of the carcinogenic agent azoxymethane (AOM; Sigma–Aldrich). Subsequently, intestinal tumor formation was promoted by three cycles of the proinflammatory reagent dextran sodium sulfate (DSS; MP Biomedicals, Santa Ana, CA, USA) in the drinking water (2% w/v). Each cycle lasted 7 days with 14 days of recovery in between. After one week of recovery, subsequent to the last DSS cycle, animals were randomly divided into four groups (*n* = 5 per group) and treated as follows:

Group I: daily by oral gavage with 25 mg/kg of the BCL-XL inhibitor A-1331852, dissolved in 60% Phosal 50 PG (Lipoid, Ludwigshafen, Germany), 27.5% polyethylene glycol 400 (Sigma–Aldrich), 10% ethanol, and 2.5% dimethyl sulfoxide (DMSO; Serva, Heidelberg, Germany) and with a formulation as described^35^. Group II: three times a week with 30 mg/kg 5FU, dissolved in saline, by intraperitoneal injection. Group III: with the combination of A-1331852 and 5FU. Group IV: with the respective solvents as a control. For evaluation of diarrhea severity and anal bleeding during tumor induction and treatment, a score was used as previously described^25^. After 14 days of treatment, blood was collected from the retrobulbar venous sinus and mice were sacrificed by cervical dislocation with subsequent opening of their bowel cavity. Colons were removed, rinsed with PBS and opened longitudinally. Tumors were counted and tumor diameters were measured with a sliding caliper by two blinded investigators (ALS and NS). Tumor volumes were calculated from the measured diameters with the following equation: $\frac{4}{3} \ast \pi \ast r^3$. The blood was transferred to the Diagnostic Center of the Heidelberg University Hospital for compilation of a small hemogram, according to standard procedures.

### Immunohistochemistry

Mouse colon tissues were isolated, rinsed with PBS, covered with OCT mounting medium (Science Services, Munich, Germany) and gradually frozen in the gas phase of liquid nitrogen. Eight-micrometer cryosections were cut (Cryostat, Thermo Fisher Scientific) and fixed in 4% paraformaldehyde (PFA), followed by heat induced antigen retrieval with citrate buffer (pH6). Subsequently, staining was performed by using the NovoLink Polymer Detection System (Leica Microsystems, Wetzlar, Germany), according to the manufacturer´s protocol. The following primary antibodies were used for murine tissues: BCL-XL (# 2764, Cell Signaling Technology), Ki67 and cleaved PARP (# ab16667 and # ab32064, Abcam, Cambridge, UK).

Paraffin-embedded human tissue sections were dewaxed and rehydrated using xylene and a series of graded alcohols, followed by antigen retrieval and staining with antibodies against BCL-XL (# 2764, Cell Signaling Technology) or cleaved PARP (#5625, Cell Signaling Technology), as described above.

Staining intensity was evaluated blinded for the groups by two independent and experienced examiners by utilizing a scoring system in which values for staining quantity (1–10% = 1, 11–50% = 2, 51–80% = 3, 81–100% = 4) and quality (unstained = 0, weak = 1, moderate = 2, strong = 3) were allocated and multiplied in the end^36^. Negative controls were generated by omitting the primary antibody.

### xCELLigence real-time cell analysis

The xCELLigence real-time cell analysis (RTCA) instrument (ACEA Biosciences, San Diego, CA, USA) allows continuous monitoring of cell proliferation and viability by measuring electrical impedance. Therefor, 7500 CCD 841 CoN cells in 100 µl medium per well were seeded in gold-plated 96-well E-Plates (ACEA Biosciences). Subsequently, cells were monitored with a time interval of 5 min for a total of 72 h. 24 h after seeding, cells were treated as indicated and curves were normalized at this point. Impedance was calculated by the RTCA software and reported as Cell Index (CI), as described previously^37^.

### Patients and human tissue culture

Primary CRC tissues (*n* = 27) were obtained by surgical resection in the Department of General and Transplantation Surgery, University of Heidelberg, Germany. The usage of patient tissue for research purposes was approved by the local ethics committee of the University Hospital of Heidelberg. All analyses were done anonymously and written informed consent was obtained from all donors (S-443/2013 and S-649/2012). This study was conducted in accordance with the Declaration of Helsinki. The cohort included patients diagnosed in all UICC stage (I–IV), 70% of the tumors were colon carcinomas and 30% rectal carcinoma.

Human primary CRC tissues were processed according to the protocol of Georg Gdynia and colleagues^36^. In brief, tumor tissues were cut in Krebs–Henseleit buffer into 300 µm thick slices using a vibratome (Leica Biosystems, Wetzlar, Germany), according to manufacturer´s protocol. Afterwards, tissue specimens were transferred onto filter membrane inserts and placed in six-well plates (both Corning, Corning, NY; USA). From below, wells were gently filled with about 1.5 ml DMEM culture medium (Gibco), supplemented with 10% heat-inactivated fetal calf serum (PAA Laboratories), 1% penicillin/streptomycin (PAA Laboratories) and the respective inhibitor or chemotherapeutic agent as indicated, until the lower surface of the membrane was reached. Tissue specimens were kept at the air-liquid interface for 48 h in a humidified atmosphere at 37 °C and 5% CO_2_. Finally, tissue slices were fixed in 10% formalin and paraffin embedded. Two-micrometer sections were stained with Hematoxylin and Eosin (H&E) for blinded assessment of tissue viability by a trained pathologist (GG).

### Statistical analysis

Statistical analysis was performed using R (version 3.5.1; r-project.org) and GraphPad Prism 8.2.1 (GraphPad; San Diego, CA, USA). All in vitro experiments were performed in at least triplicates and data are presented as mean + SD. Obtained data were submitted to analysis of variance (ANOVA) with the post-hoc Tukey multiple comparison test or by two-way repeated measures analysis of variance (two-way ANOVA) followed by Dunnett´s multiple comparison test. *P* values < 0.05 were considered significant and were indicated as following: *P < 0.05, **P < 0.01, ***P < 0.001.

## Results

### BCL-XL is highly active in colorectal cancer

Pan-cancer RNA sequencing cohorts enable the comparative transcriptomic analysis of biological networks, pathways, and genes of interest. Since mRNA levels not necessarily correlate with protein activity, we used the metaVIPER algorithm to infer the activity of antiapoptotic BCL-2 proteins from RNA sequencing data. This algorithm was designed to assess the activity of a protein by an integrative analysis of its transcriptional targets, i.e., its interactome^29^. In this way, RNA sequencing data cannot only reveal changes on the transcriptional level but also posttranslational effects.

**Fig. 1:**
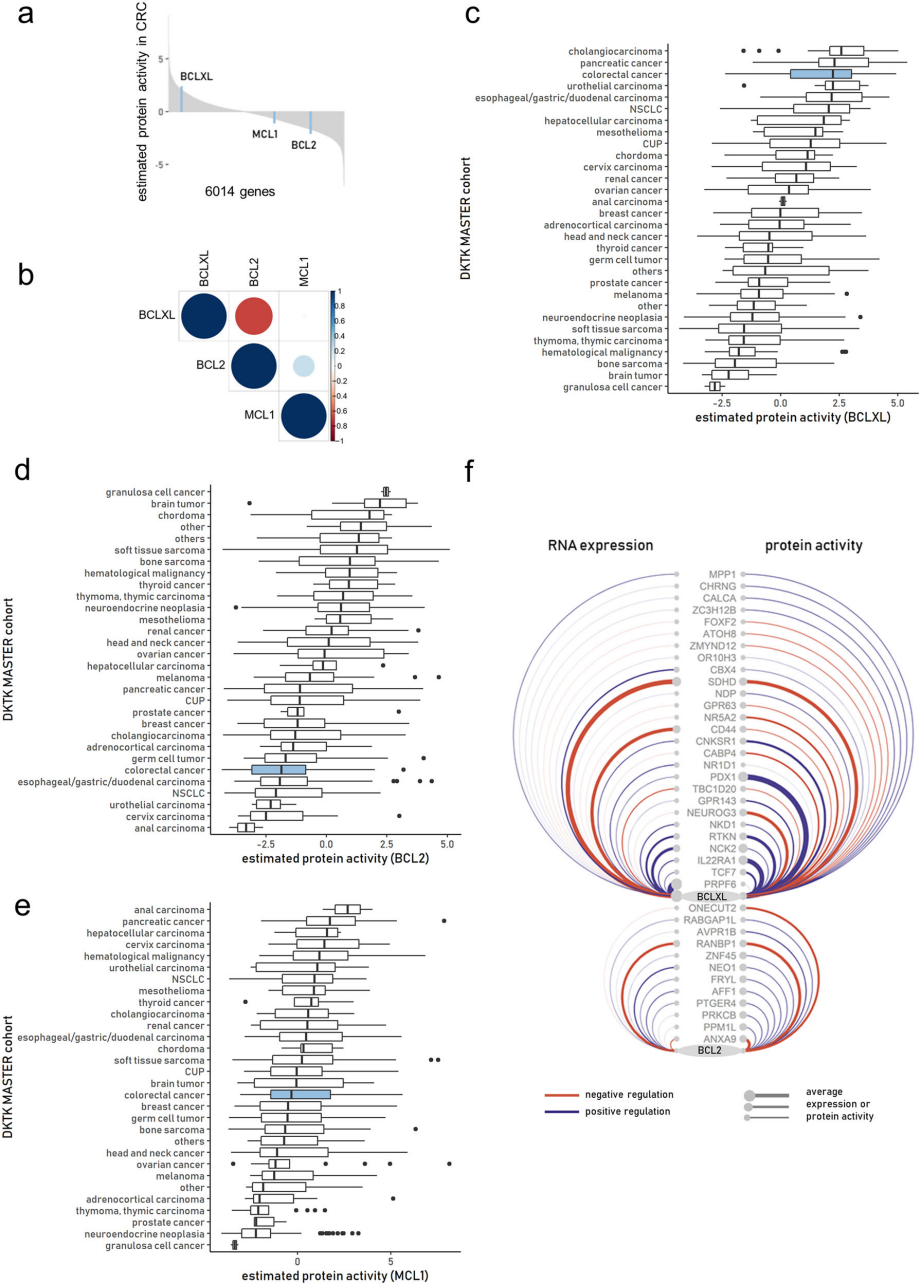
Pan-cancer protein activity of BCL-XL, BCL-2, and MCL-1. **a** Waterfall plot of the ranked 6014 master regulators identified in the CRC cohort (*n* = 68) by metaVIPER algorithm. **b** Heatmap of correlation coefficients between estimated protein activities of BCL-XL, BCL-2, and MCL-1 across the CRC cohort (*n* = 68). **c**–**e** Ranking of tumor entities across the NCT/DKTK MASTER cohort (*n* = 1521) according to the estimated median protein activity of BCL-XL (**c**), BCL-2 (**d**), and MCL-1 (**e**) obtained by metaVIPER algorithm. **f** Arc diagram of the regulatory network of BCL-XL and BCL-2. The edges and nodes were color-coded according the direction of regulation and weighted according the average expression or protein activity in the CRC subset of the NCT/DKTK MASTER cohort.

Comparing the estimated protein activity of BCL-2, BCL-XL, and MCL-1 within a CRC cohort (*n* = 68) revealed only BCL-XL but not BCL-2 or MCL-1 to be highly active (Fig. 1a). Moreover, protein activity of BCL-2 was inversely correlated with that of BCL-XL (r = −0.68), while there was no correlation between MCL-1 and BCL-XL (Fig. 1b). Estimating the protein activity of BCL-2, BCL-XL, and MCL-1 within the entire NCT/DKTK MASTER cohort (*n* = 1521) showed distinct differences between entities, with CRC showing one of the highest activities of BCL-XL (Fig. 1c). In contrast, estimated protein activities of BCL-2 and MCL-1 were relatively low in CRC compared to the complete cohort (Fig. 1d, e).

Comparing RNA expression (transcripts per million, tpm), and not estimated protein activity, across the cohort, BCL-XL again ranked highest among the antiapoptotic BCL-2 proteins investigated in CRC. Intriguingly, this rank did not match the estimated protein activity, since it showed a comparably lower transcript level, indicating a posttranslational effect (Fig. S1).

The cause for the discrepancy between the expression and estimated protein activity of the BCL-2 proteins can be visualized by the weight of positive and negative regulators in the CRC subset of the MASTER cohort (Fig. 1f). By incorporating the information about the interactome, more regulators are identified that ultimately influence the protein activity of BCL-XL and BCL-2.

### Inhibition of BCL-XL induces apoptosis in human colorectal cancer cells

**Fig. 2:**
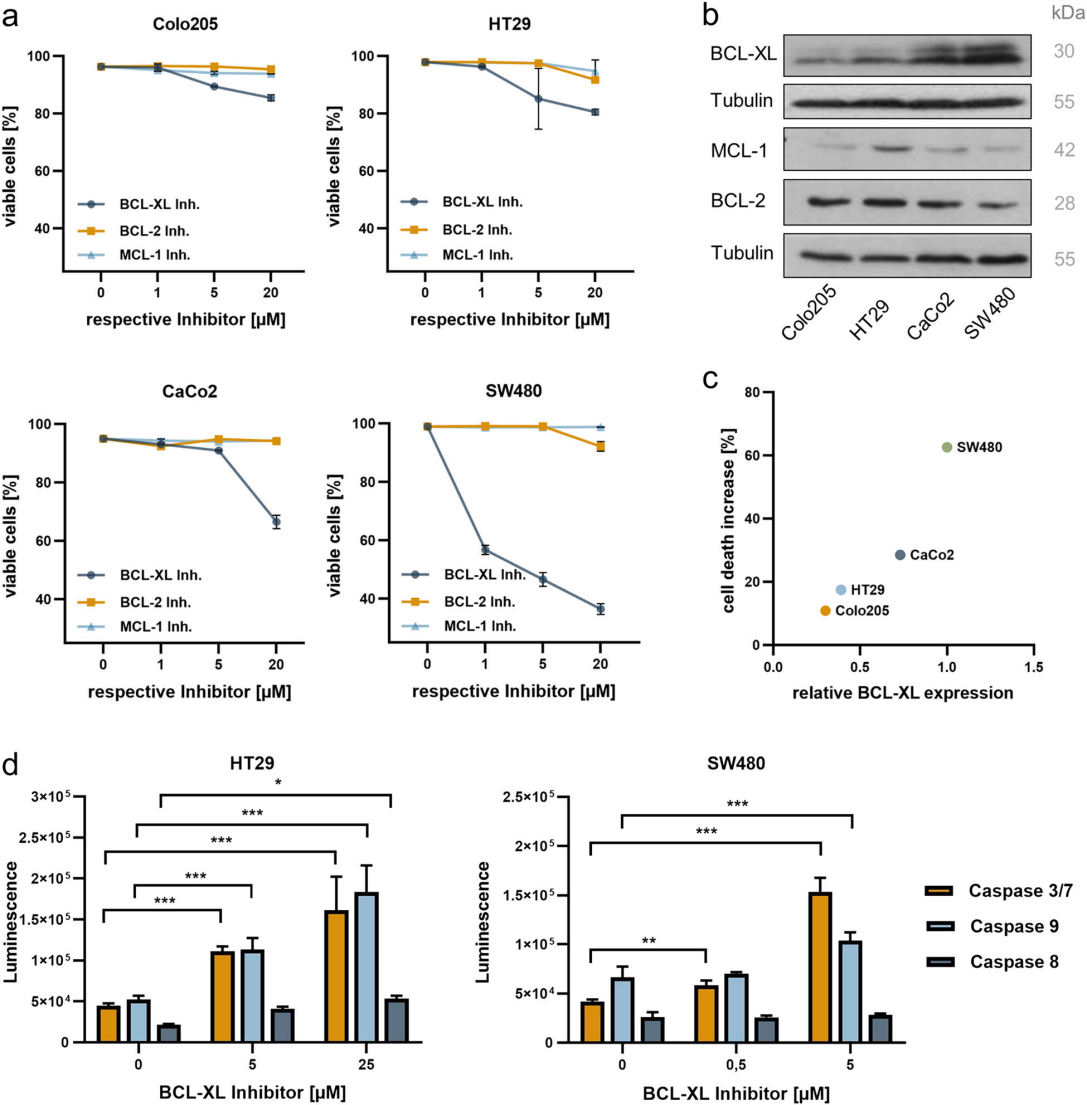
Selective BCL-XL inhibition induces apoptotic cell death in human CRC cells. **a** Human colorectal cancer cell lines Colo205, HT29, CaCo2, and SW480 were treated with the indicated concentrations of WEHI-539 (BCL-XL inhibitor), S63845 (MCL-1 inhibitor), or ABT-199 (BCL-2 inhibitor) for 48 h. Cell death was quantified by FACS analysis and is depicted as percent of surviving cells. Results are shown as mean ± standard deviation; *n* = 3. **b** Western blot analysis of whole-cell lysates from human CRC cell lines, detecting basal expression levels of antiapoptotic proteins BCL-XL, MCL-1, and BCL-2. **c** Correlation between basal BCL-XL expression, determined by densitometric analysis of **b**, and cell death increase under treatment with 20 µM WEHI-539 for 48 h. **d** Quantification of luminescence as indicator for caspase activity in WEHI-539 treated HT29 and SW480 cells. Results are shown as mean with standard deviation; *n* = 4.

In order to evaluate the efficiency of target-specific BH3-mimetics in inducing CRC cell death, four human CRC cell lines were treated with WEHI-539 (BCL-XL inhibitor), ABT-199 (BCL-2 inhibitor), and S63845 (MCL-1 inhibitor) in three different concentrations (Fig. 2a). Strikingly, all tested cell lines were most sensitive towards inhibition of BCL-XL ([Colo205: WEHI-539: 1 µM *p* = ns, 5 µM and 20 µM *p* < 0.0001; ABT-199: all concentrations *p* = ns; S63845: 1 µM *p* = ns, 5 µM *p* = 0.0002, 20 µM *p* < 0.0001]; [HT29: WEHI-539: 1 µM *p* = ns, 5 µM *p* = 0.0004, 20 µM *p* < 0.0001; ABT-199 and S63845: all concentrations *p* = ns]; [CaCo2: WEHI-539: 1 µM *p* = 0.0298, 5 µM and 20 µM *p* < 0.0001; ABT-199 and S63845: all concentrations *p* = ns] and [SW480: WEHI-539: 1 µM, 5 µM and 20 µM *p* < 0.0001; ABT-199: 1 µM and 5 µM *p* = ns, 20 µM *p* < 0.0001; S63845: all concentrations *p* = ns]). However, the degree of cell death induction varied greatly among the different cell lines. Subsequent western blot analyses with antibodies detecting BCL-XL, MCL-1, and BCL-2 revealed that susceptibility to BCL-XL inhibition strongly depends on its basal expression level, showing a linear correlation with R^2^ = 0.91 (Fig. 2b, c). Neither for MCL-1 nor for BCL-2 such a correlation was observed (Fig. S2).

To evaluate the mode of cell death induced by BCL-XL inhibition, luminescence assays measuring the activation of Caspases 3/7, 8, and 9 were done (Fig. 2d). These experiments were performed in SW480, representing a cell line with high BCL-XL expression and inhibitor susceptibility, as well as in HT29 cells, representing a cell line with low expression of BCL-XL. In both cell lines, the luminescence substrate assays revealed a dose-dependent increase of Caspase 9 and Caspase 3 activation and a minor but in HT29 cells still significant activation of Caspase 8. Since Caspase 8 is not only activated in the extrinsic pathway but can also be cleaved by Caspase 3 in a feed-forward loop, enhancing apoptosis signaling^38,39^, the observed combination indicates apoptosis induction via the intrinsic pathway.

### Inhibition of BCL-XL significantly enhances the effectiveness of chemotherapeutic agents in vitro

**Fig. 3:**
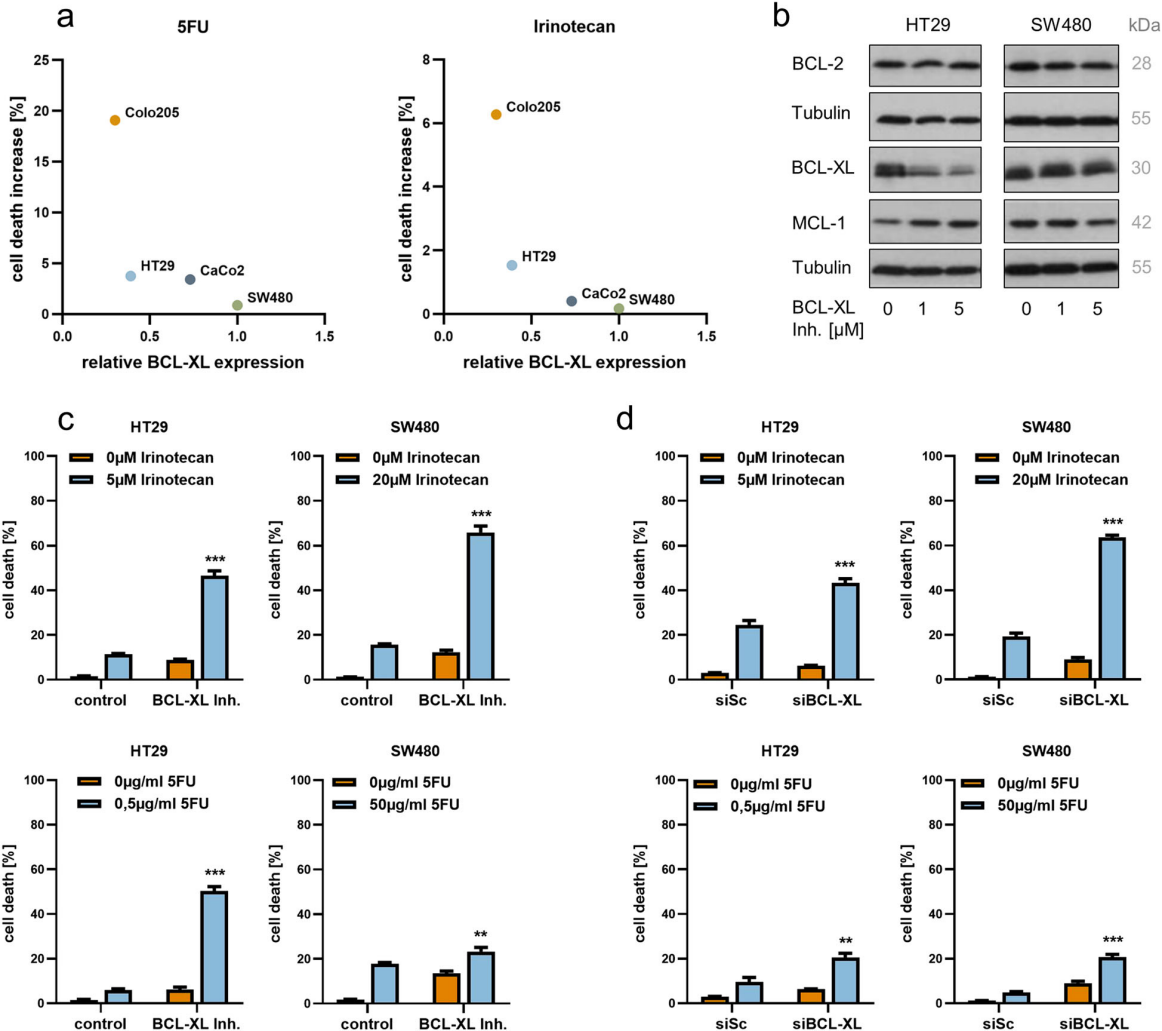
Selective BCL-XL inhibition significantly augments 5FU and irinotecan in inducing CRC cell death in vitro. **a** Correlation between basal BCL-XL expression (densitometric analyses from Fig. 2b) and cell death increase under treatment with 1 µg/ml 5-fluorouracil (5FU; left graph) or 1 µM irinotecan (right graph) for 48 h. **b** Western blot analysis of whole-cell lysates from HT29 and SW480 cells, detecting expression levels of antiapoptotic proteins BCL-XL, MCL-1, and BCL-2 after treatment with the indicated WEHI-539 concentrations for 48 h. **c** Relative quantification of cell death by FACS analysis after treatment of HT29 and SW480 cells with adjusted concentrations of WEHI-539 and/or chemotherapeutic agents irinotecan (upper panel) and 5FU (lower panel) for 48 h. The following doses have been applied. HT29: 2 µM WEHI-539, 5 µM irinotecan, 0.5 µg/ml 5FU; SW480: 0.5 µM WEHI-539, 20 µM irinotecan, 50 µg/ml 5FU. **d** Relative quantification of cell death by FACS analysis after transfection of HT29 and SW480 cells with 80 nM siRNA targeting BCL-XL and/or chemotherapeutic agents irinotecan (upper panel) and 5FU (lower panel) for 48 h. A nontargeting scrambled siRNA served as control and 5FU; irinotecan concentrations were the same as in **c**. Results in **c** and **d** are shown as mean with standard deviation; *n* = 3; **p < 0.01; ***p < 0.001.

To test the response under treatment with chemotherapeutic agents 5-fluorouracil (5FU) and irinotecan, which are commonly used in treatment regimens for CRC patients, the four CRC cell lines were incubated with equal doses of the respective cytostatic drug and cell death was subsequently quantified by FACS analysis. In this setting, the resulting cell death increase was inversely correlated with basal expression of BCL-XL, with similar results for 5FU (R^2^ = 0.79, log-transformed predictor) and irinotecan (R^2^ = 0.93, log-transformed predictor), even though the two chemotherapeutics employ different modes of action in the context of cell death induction (Fig. 3a). Since a relatively high basal BCL-XL expression level seemed to render cells susceptible towards inhibitor treatment and resistant towards chemotherapy, we combined these two treatment strategies in HT29 and SW480 cells. For both cell lines, inhibitor doses as well as cytostatic drug concentrations were individually adjusted to induce a moderate cell death increase of 5–15% under mono-drug treatment. Strikingly, the additional chemical inhibition of BCL-XL significantly enhanced the effectiveness of irinotecan (Fig. 3c; upper panels; *p* < 0.0001 for HT29 and SW480) and 5FU (lower panels; *p* < 0.0001 for HT29 and *p* = 0.0015 for SW480) in both cell lines. Western blot analyses of BCL-XL, MCL-1, and BCL-2 expression levels under WEHI-539 treatment showed decreased expression of BCL-XL in HT29 cells in presence of the inhibitor (Fig. 3b). Nevertheless, HT29 cells strongly responded to combined treatment with BCL-XL inhibitor and chemotherapy. In SW480 cells, no differences in the expression levels of the mentioned antiapoptotic proteins were observed under inhibitor treatment. In order to prove target specificity, the combination experiment was repeated applying siRNA-mediated BCL-XL (*BCL2L1*) silencing instead of chemical inhibition. Effectiveness of transfection was validated by western blot analysis (Fig. S2b). Again, a significantly increased response to chemotherapeutic agents irinotecan (Fig. 3d; upper panels) and 5FU (lower panels) was observed in both cell lines upon knockdown of BCL-XL.

### Chemical inhibition of BCL-XL is effective in the treatment of colorectal tumors in vivo

In order to examine whether colorectal tumors respond to a systemically applied BCL-XL inhibitor, a chemically induced CRC mouse model was utilized. Specifically, we aimed to compare responses to BCL-XL inhibitor treatment with responses to 5FU, which is an essential part of standard of care treatment regimens for CRC, and whether addition of a BCL-XL inhibitor can enhance the effectiveness of 5FU, as observed in vitro. Furthermore, we sought to explore the safety and toxicity profile of such a combination therapy in vivo. For the mouse experiments A-1331852, which is a WEHI-539 related but orally available BCL-XL inhibitor, was used.

**Fig. 4:**
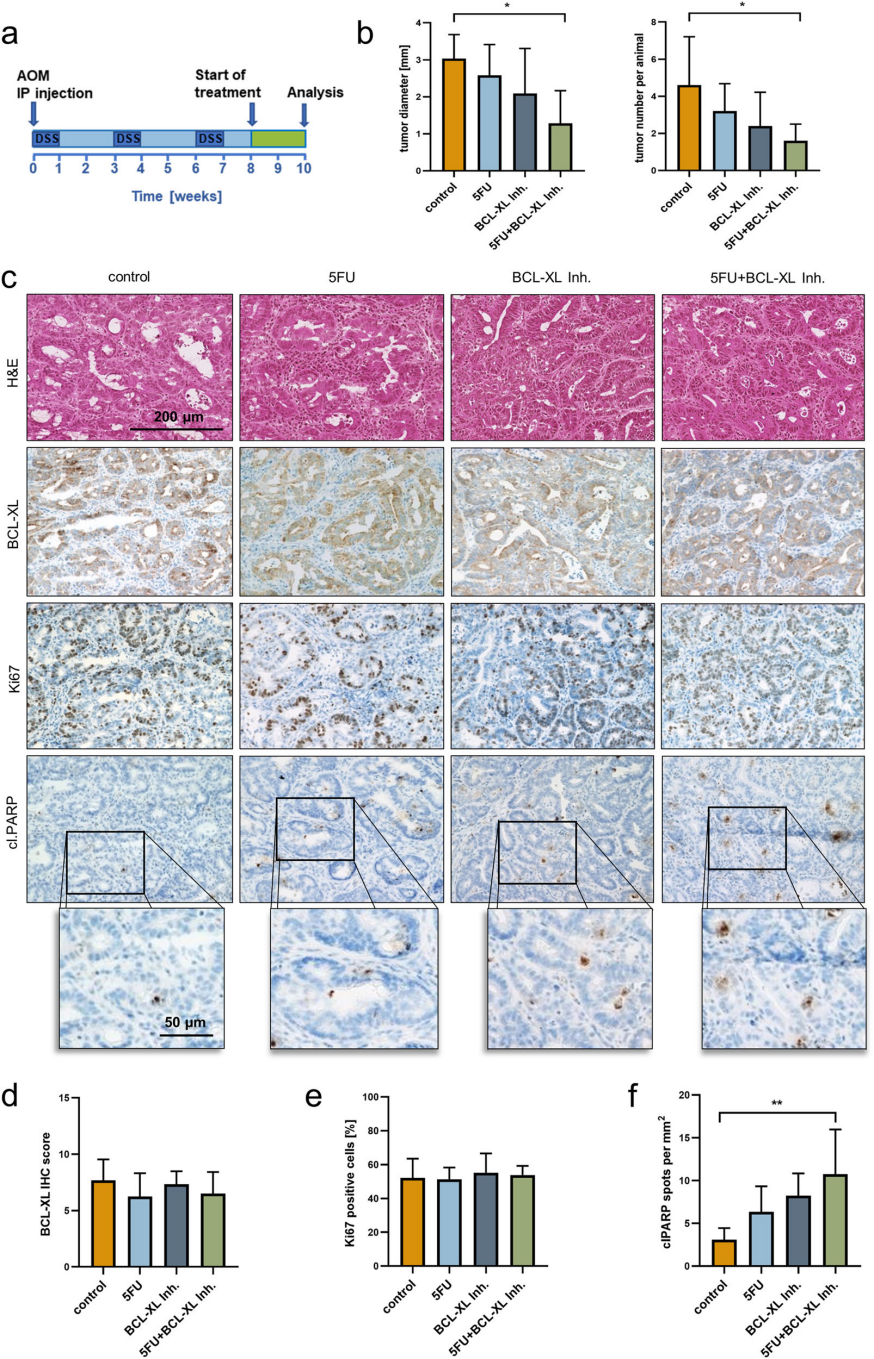
Selective BCL-XL inhibition is effective in the treatment of tumors in a murine CRC model. **a** Schematic time course with a tumor initiation and a treatment phase. Initiation: intraperitoneal injection of azoxymethane (AOM) at the start day and three cycles of dextran sodium sulfate (DSS) in the drinking water (2% w/v). Treatment: 25 mg/kg A-1331852 (orally available BCL-XL inhibitor) daily by oral gavage or 3x per week 30 mg/kg 5FU by intraperitoneal injection for a total time period of 14 days. **b** Tumor sizes (left graph) and numbers (right graph) in mice after treatment with A-1331852, 5FU, the combination of both or the respective solvents as a control (*n* = 5 animals per group). **c** Hematoxylin and Eosin (H&E) staining (upper panel) and immunohistochemical staining against BCL-XL, Ki67 (proliferation marker) and cleaved PARP (cl.PARP; apoptosis marker) on tumors derived from animals treated as depicted in **a**. Scale bars as indicated **d** Scoring of the IHC staining of BCL-XL depicted in **c**. **e** Percentage of Ki67-positive cells, referred to Hematoxylin stained nuclei. **f** Number of cl.PARP positive spots per mm^2^. Results in **b**, **d**, **e,** and **f** are shown as mean with standard deviation; *p < 0.05, **p < 0.01.

For induction of colorectal tumors, the combination of AOM and DSS was administered^40^. Subsequently, mice received either the BCL-XL inhibitor (25 mg/kg, daily by oral gavage), 5FU (30 mg/kg, 3× per week by i.p. injection), the combination of both or solely the respective solvents as a control for 2 weeks, and afterwards tumor numbers and diameters were determined. Measurement of tumor diameters revealed that both, treatment with 5FU (mean tumor diameter: 2.58 mm) as well as treatment with the inhibitor (Ø 2.09 mm), led to apparent, albeit not significant tumor shrinkage compared to controls (Ø 3.04 mm). However, the strongest effect was observed upon combined treatment. With a mean tumor diameter of 1.3 mm (Fig. 4b; left graph; *p* = 0.021) we observed in this group a significant decrease of tumor volume to 14%, if compared to controls (calculated tumor volumes are depicted in Fig. S3a). Furthermore, animals that received either 5FU or BCL-XL inhibitor treatment displayed lower tumor numbers than the controls. Adding the BCL-XL inhibitor to 5FU caused the strongest effect, proving the value of BCL-XL inhibition in combination with the fluoropyrimidine (Fig. 4b; right graph; *p* = 0.047).

To investigate whether BCL-XL expression is decreased in tumors derived from mice that received the inhibitor alone or the combination therapy, immunohistochemical staining for BCL-XL was performed (Fig. 4c). The staining revealed an unaltered expression of BCL-XL in these two groups compared to the control and the 5FU treated groups (Fig. 4d). In a second step, we sought to analyze if the observed differences in tumor size and numbers in the different groups rely on an altered proliferation activity or on cell death induction in the tumors. While we found the level of Ki67-positive cells unalteredly high (Fig. 4c, e), staining for cleaved PARP (cl.PARP), a Caspase substrate, revealed considerable apoptosis induction in tumors derived from treated animals but not in the controls. The highest cl.PARP positivity was present in tumors derived from the group that underwent combinatorial treatment with 5FU and the BCL-XL inhibitor (Fig. 4c, f; *p* = 0.006).

### Chemical inhibition of BCL-XL does not affect nontransformed intestinal epithelial cells

**Fig. 5:**
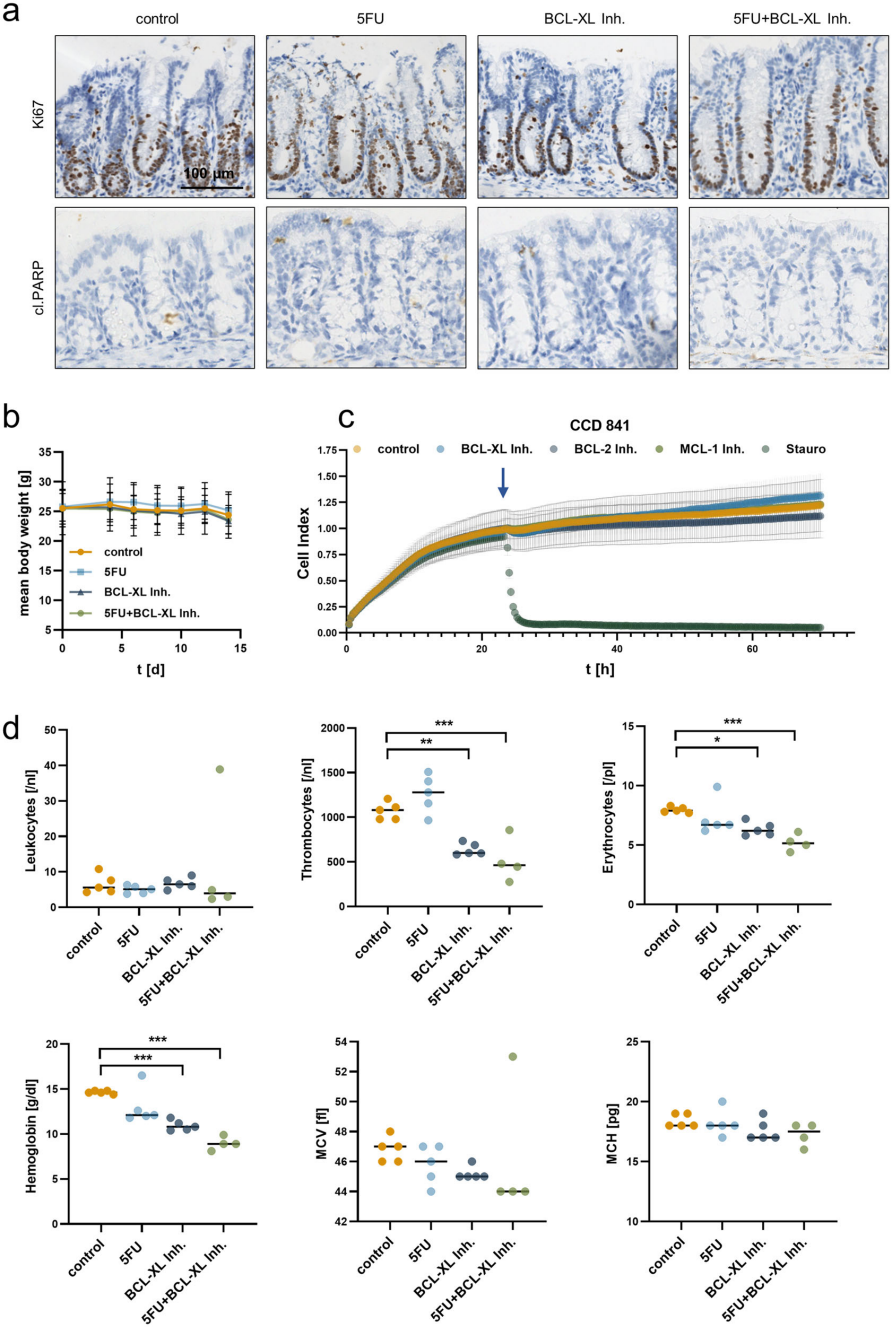
Selective BCL-XL inhibition does not induce cell death in intestinal epithelial cells. **a** Immunohistochemical staining against Ki67 (proliferation marker) and cleaved PARP (cl.PARP; apoptosis marker) on longitudinal sections of colon crypts, derived from animals treated as depicted in (4a). Scale bar indicates 100 µm. **b** Body weight alterations during the course of treatment. **c** Impedance monitoring of human intestinal epithelial cell line CCD 841 CoN for 72 h. After 24 h (blue arrow), cells were treated with 20 µM of WEHI-539 (BCL-XL inhibitor), S63845 (MCL-1 inh.) or ABT-199 (BCL-2 inh.). DMSO was used as vehicle control and 2 µM Staurosporine (Stauro) as positive control for cell death induction. Results are shown as mean ± standard deviation; *n* = 3. **d** Small hemogram from mice after indicated treatment. MCV mean corpuscular volume, MCH mean corpuscular hemoglobin. Results are shown as median; *p < 0.05; **p < 0.01; ***p < 0.001.

By immunohistochemical staining of tumor-free mucosa with antibodies detecting Ki67 and cl.PARP, we found the proliferative capacity of nontransformed intestinal epithelial cells to be marginally decreased and the viability to be unaltered in mice after treatment with 5FU, the BCL-XL inhibitor or the combination of both (Fig. 5a and for quantification Fig. S3b). In addition, the chosen doses of 5FU and the inhibitor or their combined application did not induce significant weight loss during the 2 weeks of treatment (Fig. 5b). To further test whether intestinal epithelial cells are less prone to cell death induction via chemical BCL-XL inhibition compared to CRC cells, nontransformed human intestinal epithelial cells (CCD 841 CoN cell line) were treated with 20 µM WEHI-539, 20 µM ABT-199, or 20 µM S63845, representing the highest doses used for screening in human CRC cell lines (Fig. 2a). Two-micromolar Staurosporine were used to generate a positive control for cell death induction. For the measurement, the xCELLigence system that allows real-time monitoring of proliferation and viability has been used. The benefit of this system is a non-invasive, label-free and close monitoring of growth inhibition or cell death induction of adhesive cells under treatment. The device uses gold-plated microelectrodes to measure electrical impedance from which the Cell Index (CI) as unitless parameter is calculated^37^. Hereby, we found that none of the inhibitors significantly induced cell death in CCD 841 CoN cells (Fig. 5c).

To further assess putative adverse effects on blood cells, blood was collected after treatment with 5FU, the BCL-XL inhibitor, or the combination of both. We found no significant changes in leukocyte numbers; however, thrombocyte and erythrocyte counts were significantly decreased in mice treated with the BCL-XL inhibitor alone or in combination with 5FU (Fig. 5d; [the inhibitor lowered thrombocytes to 60%; *p* = 0.0026 and the combination to 48%; *p* = 0.0005]; [the inhibitor lowered erythrocytes to 80%; *p* = 0.034 and the combination to 65%; *p* = 0.001] all comparisons were made to the respective controls). Despite a known effect on hematopoiesis, 5FU alone did neither lead to a significant decrease of thrombocytes nor erythrocytes. Additional application of 5FU to BCL-XL inhibition aggravated the phenotype observed under inhibitor-only treatment. In order to analyze the reason for the decreased number of erythrocytes in further detail, we additionally determined hemoglobin levels, the average erythrocyte size (mean corpuscular volume, MCV) and the amount of hemoglobin per red blood cell (mean corpuscular hemoglobin, MCH). The analyses revealed significantly decreased overall hemoglobin levels after inhibitor treatment (inhibitor: *p* = 0.0003; combination: *p* < 0.0001) but an unaltered hemoglobin content per erythrocyte. In combination with the slightly decreased MCV values in the inhibitor-treated groups, this demonstrated the occurrence of microcytic anemia.

### Chemical inhibition of BCL-XL in combination with 5FU significantly decreases tumor cell viability in human colorectal cancer specimens ex vivo

**Fig. 6:**
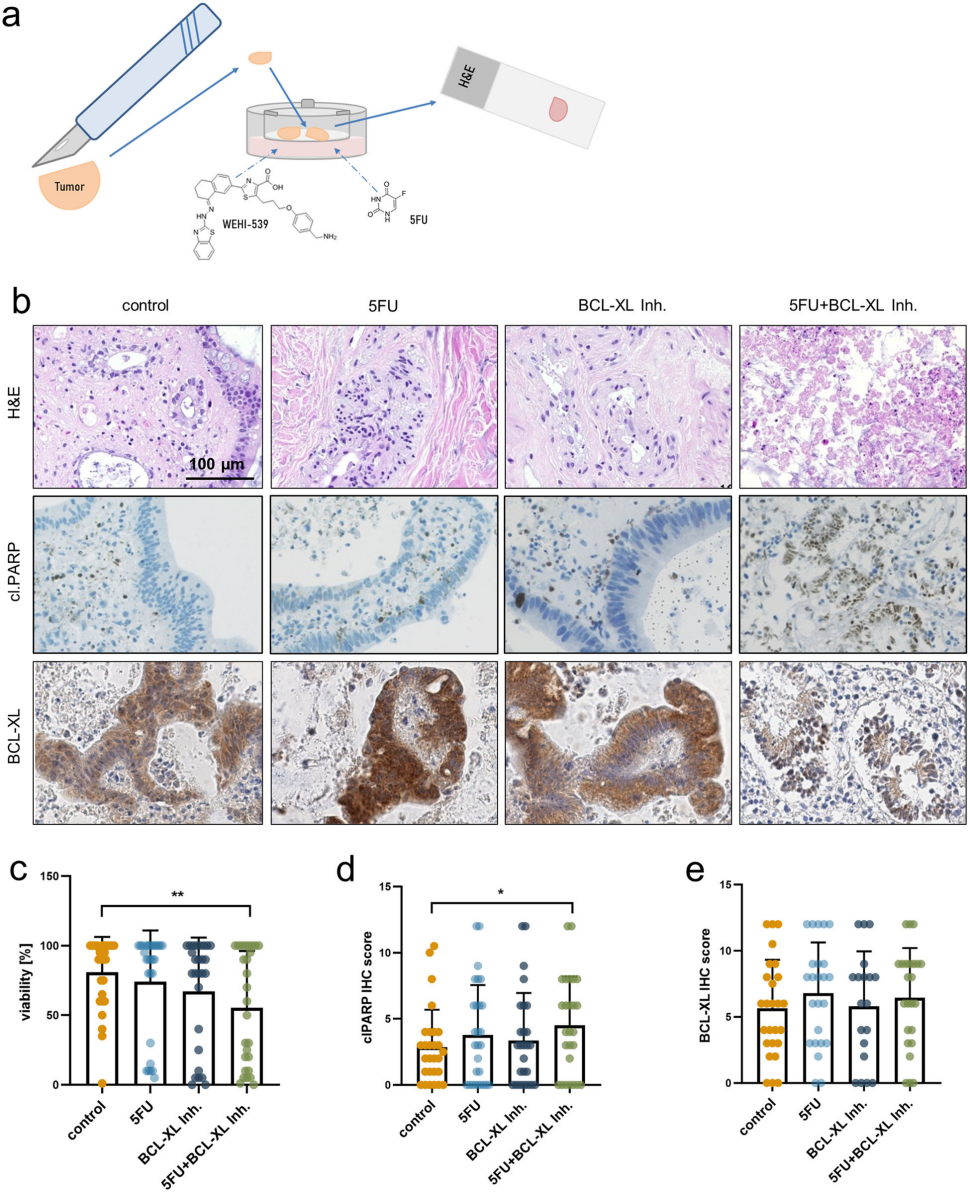
Selective BCL-XL inhibition significantly enhances the antineoplastic effect of 5FU in patient-derived CRC specimens. **a** Schematic display of *ex vivo* cultures of human CRC specimens. Tumor samples were taken upon surgical resection, cut and kept in inserts at the air-liquid interface for 48 h. Culture media were supplemented with 5 µM WEHI-539, 50 µg/ml 5FU or the respective vehicle controls. **b** Representative H&E staining (upper panel) and IHC staining against cleaved PARP (cl.PARP) and BCL-XL of patient-derived CRC specimens after treatment as depicted in **a**. Scale bar indicates 100 µm. **c** Quantification of tumor cell viability based on the H&E staining in **b**. **d** Scoring of the IHC staining of clPARP depicted in **b**. **e** Scoring of the IHC staining of BCL-XL depicted in **b**. Results in **c**–**e** are shown as individual values with boxes depicting mean values; *p < 0.05; **p < 0.01.

In order to transfer our in vitro and in vivo findings into a more translational setting, we utilized tissue cultures of patient-derived CRC specimens. To this end, CRC samples from patients who underwent surgery (*n* = 27) were freshly cut and treated for 48 h. Patient characteristics regarding age, gender, UICC stage and tumor localization are itemized in supplementary table 1. From each patient sample, several slices were taken and treated with either 50 µg/ml 5FU, 5 µM WEHI-539, the combination of both or solely with the respective solvents as a control, by supplementing the medium (Fig. 6a). H&E staining with subsequent histomorphologic analysis revealed an only minor decrease of tumor cell viability under single agent therapy but a significant loss of viability under the combinatorial treatment (Fig. 6b, c; *p* = 0.023). This finding was confirmed by immunohistochemical staining of cleaved PARP, which showed that the combination of 5FU and the BCL-XL inhibitor led to a significant increase of the apoptotic cell death level (Fig. 6b, d; *p* = 0.039). In-line with our in vivo findings, immunohistochemical staining of BCL-XL revealed no significant expression differences under treatment (Fig. 6b, e).

## Discussion

Since avoidance of apoptosis is a general hallmark of tumors, cell death preventing factors, such as antiapoptotic members of the BCL-2 protein family and their potential inhibition, are in the focus of cancer research^41^. Thus, there is a pending medical need for evaluating the effectiveness and safety of novel and selective BH3-mimetics, especially in solid tumors, which urged us to further delineate their potential in the context of CRC treatment.

Emerging concepts based on functional studies suggest a tissue and organ specific rather than redundant function of antiapoptotic Bcl-2 proteins^42–44^. Few studies have investigated the specific interactome of Bcl-2 proteins in certain tumor subtypes^43,44^.

In previous work we had identified BCL-XL as the only antiapoptotic BCL-2 protein that is overexpressed in CRC^25^. In-line, CRC shows one of the highest frequencies of *BCL2L1* (gene coding for BCL-XL) amplifications across The Cancer Genome Atlas (TCGA) database^45^. By using a network biology-based approach, we were now for the first time able to derive protein activity of BCL-2, BCL-XL, and MCL-1 in a pan-cancer cohort (NCT/DKTK MASTER cohort). Protein activity correlates with mRNA expression in most cases. Estimated protein activity by the mVIPER algorithm summarizes the expression of transcriptional targets of each protein in the regulon leading to an estimated high protein activity in a couple of cases with low mRNA expression and vice versa. We could show that CRC ranks on the third place among all investigated tumor entities regarding estimated BCL-XL activity. Interestingly, a ranking of tumor entities across the pan-cancer cohort according to transcript levels of BCL-XL led to a slightly different positioning of CRC. This could point to a stabilization of the protein in CRC. Since a decreased expression of proapoptotic members of the BCL-2 protein family would have a similar functional outcome, this effect could also be caused by a shift in the balance between anti- and proapoptotic proteins towards enhanced BCL-XL activity^46^. This finding underlines the usefulness of estimating protein activity in addition to RNA-seq.

In-line with the above mentioned findings, human CRC cell lines were found to be highly sensitive towards selective inhibition of BCL-XL but not MCL-1 or BCL-2, even though binding affinity of WEHI-539 is with a dissociation constant (K_D_) of 0.6 nM lower than that of S63845 (K_D_ = 0.19 nM)^47,48^. Noticeably, we found that the susceptibility towards WEHI-539 treatment correlated with the basal expression level of BCL-XL. This coincides with a previous study utilizing various human CRC cell lines^45^. Since increased expression of antiapoptotic BCL-2 proteins induces therapy resistance in a variety of human tumors^49–51^, we sought to evaluate whether basal BCL-XL levels might influence the response to the standard-of-care chemotherapeutic agents 5FU and irinotecan. For both drugs, we found a similar pattern and an inverse correlation of BCL-XL expression and cell death in response to chemotherapeutic treatment. Taken together, these findings point to a potential role of BCL-XL in chemotherapy resistance.

In order to evaluate the potential of a combined approach, we treated human CRC cells with WEHI-539, 5FU or irinotecan or combinations thereof. In all constellations, we found a significantly enhanced cell death induction under combined treatment. Lately, a direct binding of irinotecan to BCL-XL has been described as an additional mode of action besides its known function as DNA topoisomerase I inhibitor^52^. This might explain why the combination of WEHI-539 with irinotecan is even more efficient than the combination with 5FU. However, because 5FU is part of both standard regimens for the treatment of CRC (FOLFOX and FOLFIRI), we chose this therapeutic agent for further in vivo experiments. By utilizing a well-established chemically induced CRC mouse model^40^, we were able to show that the combination of 5FU and A-1331852, which is an orally available derivative of WEHI-539^35^, but not the respective mono-therapies, significantly decreased tumor size and overall tumor burden. This is in-line with a previous study by Leverson et al. in which A-1331852 was applied as single agent or in combination with docetaxel. In several subcutaneous xenograft models the authors found only modest anti-tumor activity of the BCL-XL inhibitor alone, which was however strongly enhanced in the presence of docetaxel^35^.

In contrast to tumor cells, neither the murine intestinal mucosa nor human intestinal epithelial cells were affected in their viability by BCL-XL inhibition. In mice, the good treatment tolerability was furthermore mirrored by the unchanged average body weight in the treated groups. Taken together, our data indicate that targeting BCL-XL has cancer cell specific effects while sparing the healthy epithelial cells.

The first clinical trials with the dual BCL-2/BCL-XL inhibitor ABT-263 revealed that the dose-limiting side effects are leukopenia and thrombocytopenia^53,54^. Whereas leukopenia was mainly attributed to the inhibition of BCL-2, inhibition of BCL-XL is presumably the major cause of thrombocytopenia observed upon ABT-263 treatment^35^. In-line with this, we found no significant difference in the number of leukocytes in BCL-XL inhibitor-treated mice. Although the number of thrombocytes was significantly reduced by the BCL-XL inhibitor, we found no spontaneous bleeding in the treated mice. Besides thrombocytopenia, the hemogram revealed lowered erythrocyte and hemoglobin levels after inhibitor treatment. Reduced hemoglobin levels could be caused by an apoptosis-independent role of BCL-XL, as shown in a previous study postulating a requirement of BCL-XL for heme synthesis^55^. Furthermore, BCL-XL seems to prevent cell death induction in late-stage erythroblasts^56^. Nevertheless, the adverse effects on platelet counts and hemoglobin levels seem manageable by appropriate dosing and timing of inhibitor treatment. In sum, our in vivo studies suggest that specific targeting of BCL-XL has moderate side effects on the hematopoietic system.

Translating the findings from the mouse model in a closer-to-patient setting, we treated 27 patient-derived CRC specimens with WEHI-539, 5FU or the combination thereof ex vivo. The results support our in vivo findings, because a significant decrease of tumor cell viability was only observed under combinatorial but not mono-drug treatment. Taken together, this study identifies BCL-XL among the group of antiapoptotic BCL-2 proteins as the most promising target for CRC treatment. Furthermore, we proved in vitro, in vivo and ex vivo that additional inhibition of BCL-XL augments the efficacy of 5FU. This raises the possibility to render tumor cells susceptible towards lower doses of chemotherapy or even overcome treatment resistance. In addition, we could show in the murine model that nontransformed intestinal epithelial cells stay unaffected by the inhibition of BCL-XL and that treatment-efficient inhibitor doses have a good tolerability without unmanageable adverse effects. These findings might pave the way for future clinical applications.

## Supplementary information

Supplementary Figure Legends

Supplementary Figure 1

Supplementary Figure 2

Supplementary Figure 3

Supplementary Table 1
