# Supplementary material for: Identification of BCL-XL as highly active survival factor and promising therapeutic target in colorectal cancer
Source: Cell Death Dis. 2020 Oct 17;11(10):875. doi: 10.1038/s41419-020-03092-7 (PMC7568722; doi:10.1038/s41419-020-03092-7)
Supplement: Supplementary file 1 — Supplementary Figure Legends [file 41419_2020_3092_MOESM1_ESM.docx]

**Supplementary Figure Legends**

***Figure S1: Pan-cancer RNA-Seq of BCL-XL, BCL-2 and MCL-1.* a)** Waterfall plot of the 20501 genes identified in the CRC cohort (n=68), ranked according to their transcripts per million (tpm) value. **b)** Heatmap of correlation coefficients between tpm values of BCL-XL, BCL-2 and MCL-1 across in the CRC cohort (n=68). **c-e)** Ranking of tumor entities across the NCT/DKTK MASTER cohort (n=1521) according to scaled tpm values of BCL-XL (c), BCL-2 (d) and MCL-1 (e).

***Figure S2: Lack of correlation between the basal expression of BCL-2 or MCL-1 in CRC cells and the susceptibility towards the respective inhibitor.* a)** Correlation between basal BCL-2 (left) and MCL-1 (right) expression in four human CRC cell lines (Colo205, HT29, CaCo2 and SW480), determined by densitometric analysis of Figure 2b, and cell death increase under treatment with 20 µM ABT-199 (BCL-2 inhibitor) and S63845 (MCL-1 inhibitor), respectively, for 48 h. **b)** Western blot analysis of whole cell lysates from HT29 and SW480 cells after transfection with 80 nM siRNA targeting BCL-XL for 48 h.

***Figure S3:* a)** Mean tumor volumes in mice after treatment with the BCL-XL inhibitor A-1331852, 5FU, the combination of both or the respective solvent controls (n=5 per group), based on the measured tumor diameters in (4b). **b)** Quantification of tumor cell proliferation and number of cl.PARP positive spots per mm^2^ based on the Ki67 and cl.PARP staining depicted in Figure 5a. Results are shown as mean with standard deviation; *p < 0.05.

***Supplementary Table 1:*** Overview of CRC patient characteristics.
