# Supplementary figures and images for: Identification of BCL-XL as highly active survival factor and promising therapeutic target in colorectal cancer
Source: Cell Death Dis. 2020 Oct 17;11(10):875. doi: 10.1038/s41419-020-03092-7 (PMC7568722; doi:10.1038/s41419-020-03092-7)

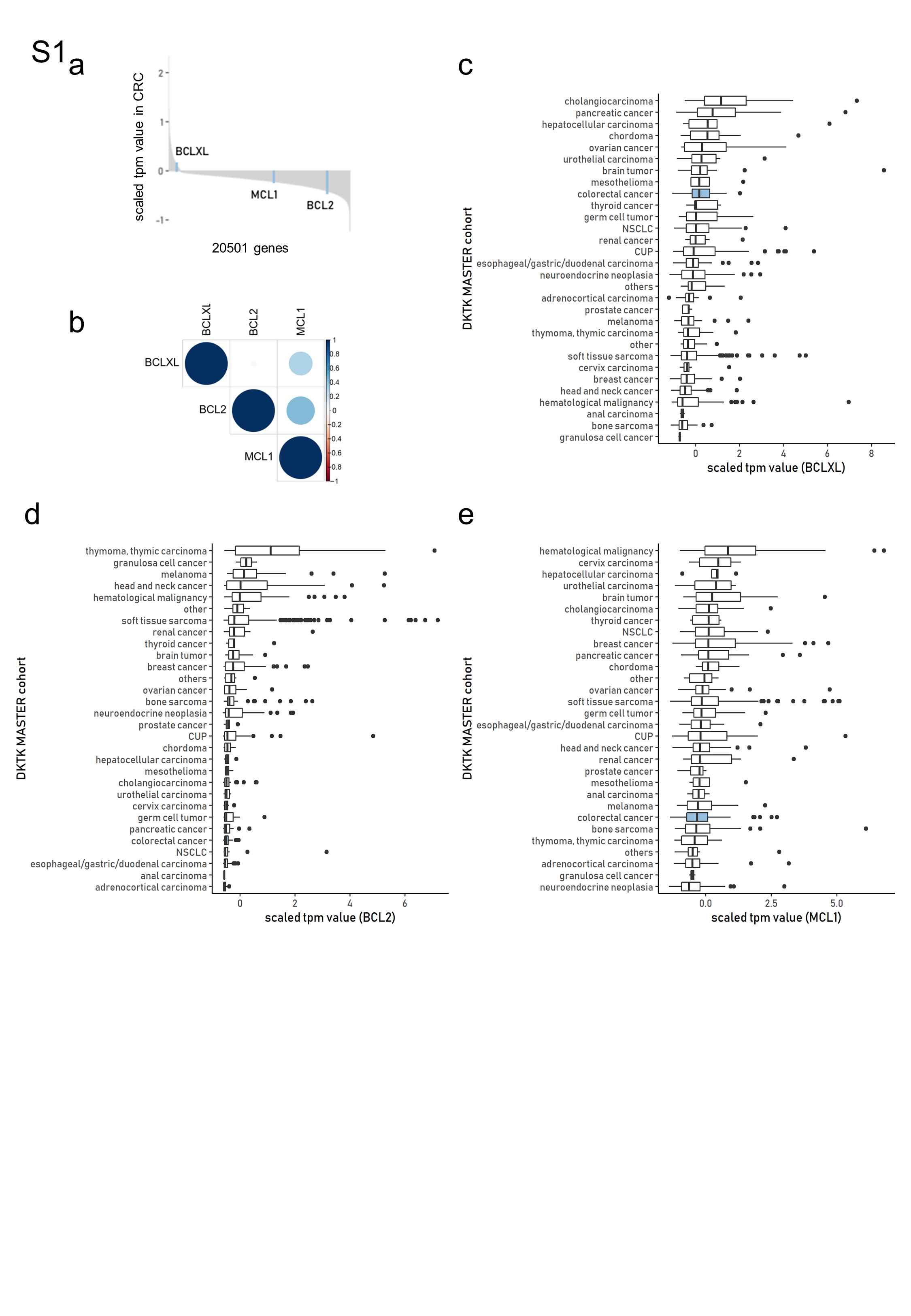

Supplement: Supplementary file 2 — Supplementary Figure 1 [file 41419_2020_3092_MOESM2_ESM.tif]

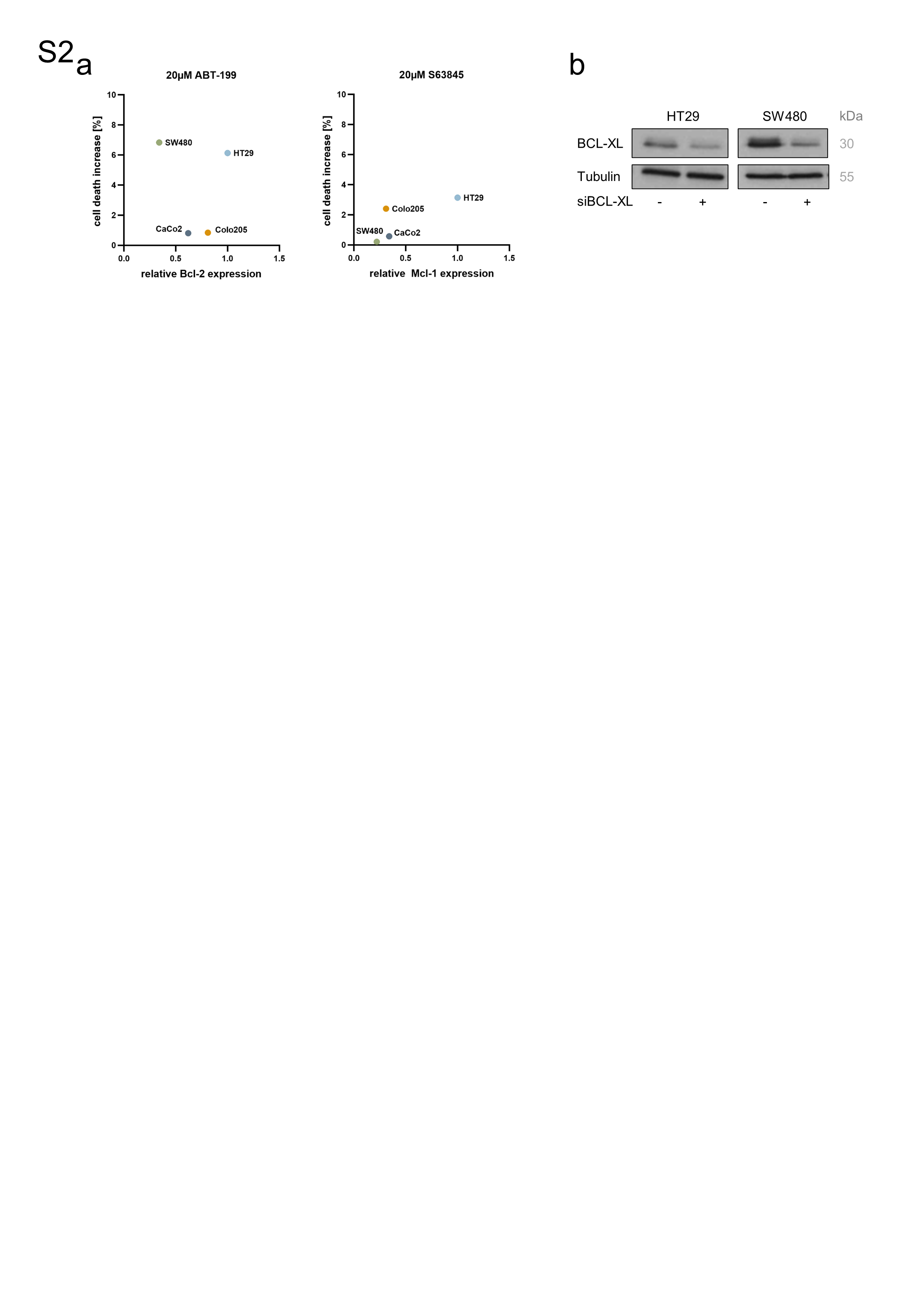

Supplement: Supplementary file 3 — Supplementary Figure 2 [file 41419_2020_3092_MOESM3_ESM.tif]

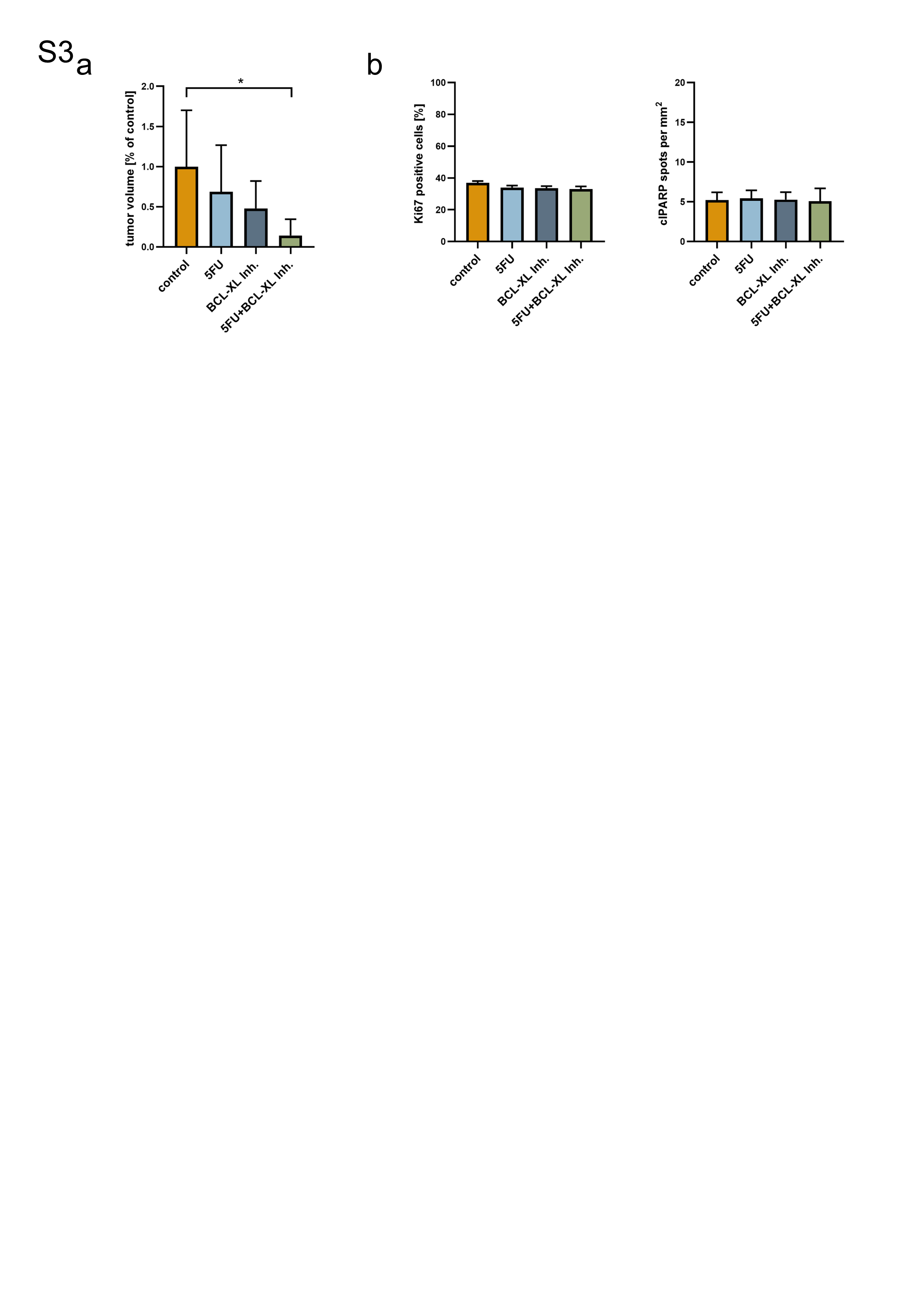

Supplement: Supplementary file 4 — Supplementary Figure 3 [file 41419_2020_3092_MOESM4_ESM.tif]

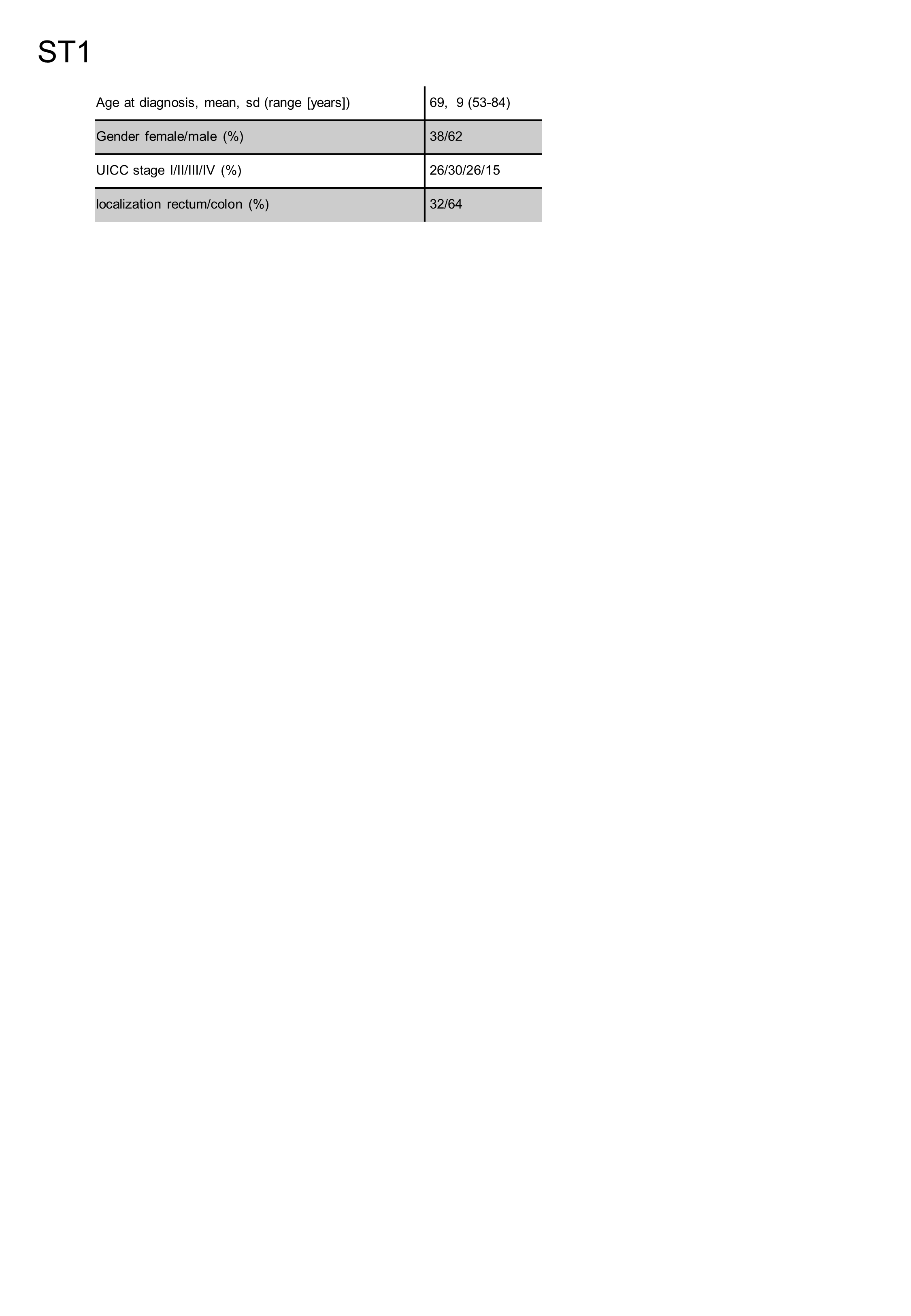

Supplement: Supplementary file 5 — Supplementary Table 1 [file 41419_2020_3092_MOESM5_ESM.tif]
